# Supplementary material for: NORFA, long intergenic noncoding RNA, maintains sow fertility by inhibiting granulosa cell death
Source: Commun Biol. 2020 Mar 18;3:131. doi: 10.1038/s42003-020-0864-x (PMC7080823; doi:10.1038/s42003-020-0864-x)
Supplement: Supplementary file 1 — Supplementary Information [file 42003_2020_864_MOESM1_ESM.pdf]

Supplementary Figures

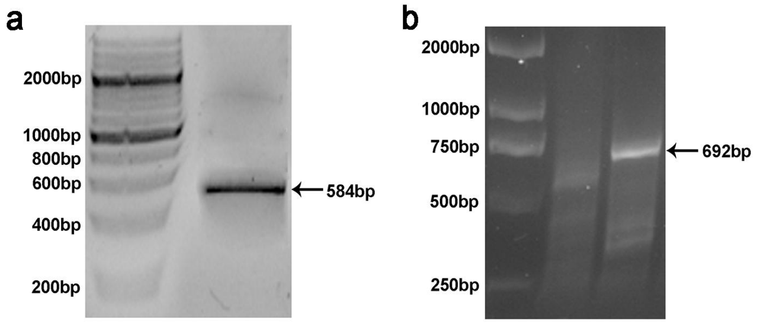

Supplementary Figure 1. 5'- and 3'-RACE analyses for porcine lincRNA-NORFA. Gel images showing the nested PCR amplified products obtained from 5'-RACE (**a**) and 3'-RACE (**b**) analyses. Black arrows indicate the products at 584 bp and 692 bp in length, respectively. DNA molecular weight markers are also represented.

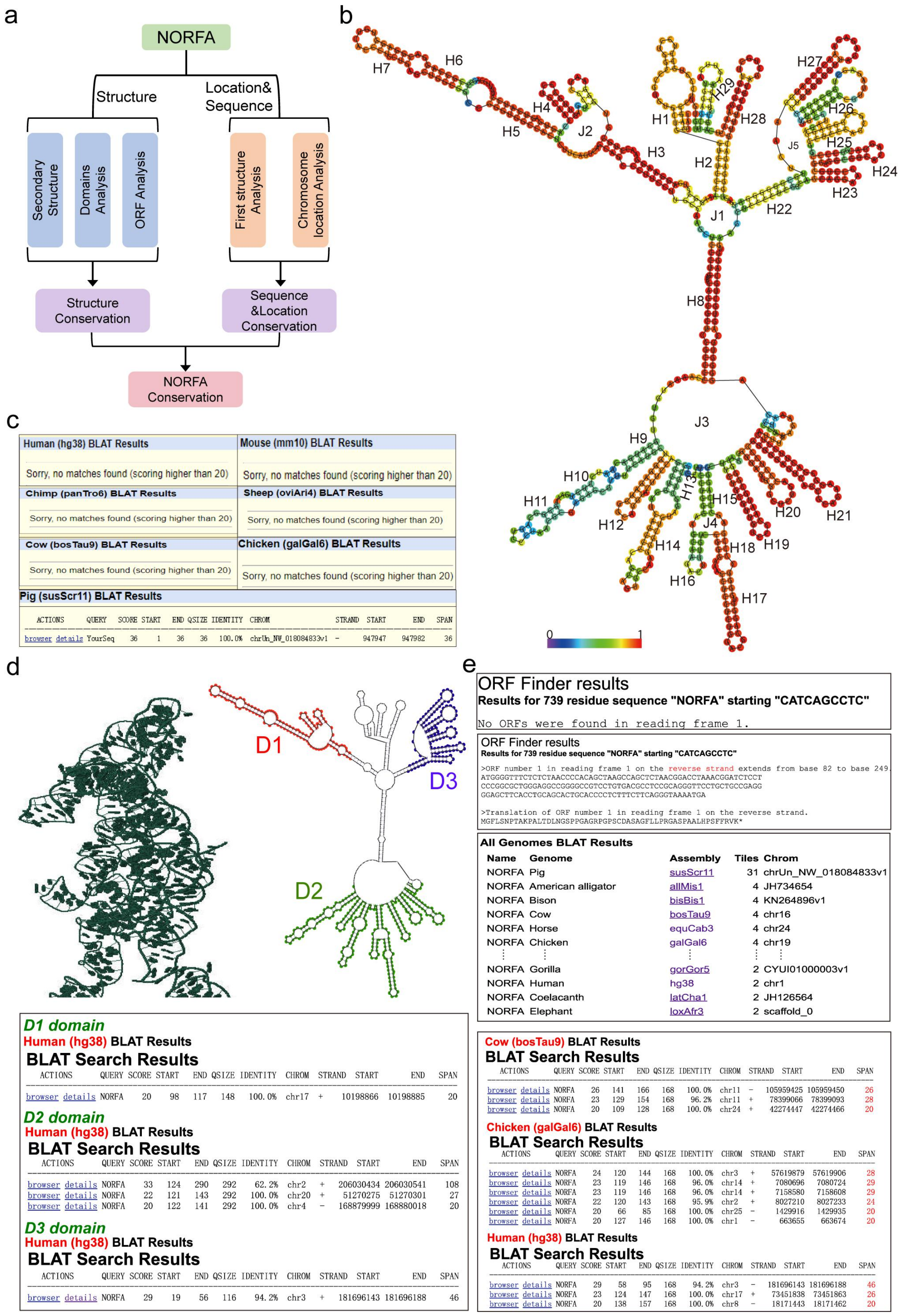

a
NORFA
Structure
Location&
Sequence
Secondary Structure
Domains Analysis
ORF Analysis
First structure Analysis
Chromosome location Analysis
Structure Conservation
Sequence &Location Conservation
NORFA Conservation
b
H1 H2 H3 H4 H5 H6 H7 H8 H9 H10 H11 H12 H13 H14 H15 H16 H17 H18 H19 H20 H21 H22 H23 H24 H25 H26 H27 H28 H29
J1 J2 J3 J4 J5
0
1
c
Human (hg38) BLAT Results
Sorry, no matches found (scoring higher than 20)
Mouse (mm10) BLAT Results
Sorry, no matches found (scoring higher than 20)
Chimp (panTro6) BLAT Results
Sorry, no matches found (scoring higher than 20)
Sheep (oviAri4) BLAT Results
Sorry, no matches found (scoring higher than 20)
Cow (bosTau9) BLAT Results
Sorry, no matches found (scoring higher than 20)
Chicken (galGal6) BLAT Results
Sorry, no matches found (scoring higher than 20)
Pig (susScr11) BLAT Results
ACTIONS QUERY SCORE START END QSIZE IDENTITY CHROM STRAND START END SPAN
browser details YourSeq 36 1 36 36 100.0% chrUn_NW_018084833v1 - 947947 947982 36
d
D1
D2
D3
D1 domain
Human (hg38) BLAT Results
BLAT Search Results
ACTIONS QUERY SCORE START END QSIZE IDENTITY CHROM STRAND START END SPAN
browser details NORFA 20 98 117 148 100.0% chr17 + 10198866 10198885 20
D2 domain
Human (hg38) BLAT Results
BLAT Search Results
ACTIONS QUERY SCORE START END QSIZE IDENTITY CHROM STRAND START END SPAN
browser details NORFA 33 124 290 292 62.2% chr2 + 206030434 206030541 108
browser details NORFA 22 121 143 292 100.0% chr20 + 51270275 51270301 27
browser details NORFA 20 122 141 292 100.0% chr4 - 168879999 168880018 20
D3 domain
Human (hg38) BLAT Results
BLAT Search Results
ACTIONS QUERY SCORE START END QSIZE IDENTITY CHROM STRAND START END SPAN
browser details NORFA 29 19 56 116 94.2% chr3 + 181696143 181696188 46
e
ORF Finder results
Results for 739 residue sequence "NORFA" starting "CATCAGCCTC"
No ORFs were found in reading frame 1.
ORF Finder results
Results for 739 residue sequence "NORFA" starting "CATCAGCCTC"
>ORF number 1 in reading frame 1 on the reverse strand extends from base 82 to base 249.
ATGGGGTTTCTCTCTAACCCCACAGCTAAGCCAGCTCTAACGGACCTAAACGGATCTCCT
CCCGGCGCTGGGAGGCCGGGGCCGTCCTGTGACGCCTCCGCAGGGTTCCTGCTGCCGAGG
GGAGCTTCACCTGCAGCACTGCACCCCTCTTTCTTCAGGGTAAAATGA
>Translation of ORF number 1 in reading frame 1 on the reverse strand.
MGFLSNPTAKPALTDLNGSPPGAGRPGPSCDASAGFLLPRGASPAALHPSFFRVK*
All Genomes BLAT Results
Name Genome Assembly Tiles Chrom
NORFA Pig susScr11 31 chrUn_NW_018084833v1
NORFA American alligator allMis1 4 JH734654
NORFA Bison bisBis1 4 KN264896v1
NORFA Cow bosTau9 4 chr16
NORFA Horse equCab3 4 chr24
NORFA Chicken galGal6 4 chr19
NORFA Gorilla gorGor5 2 CYUI01000003v1
NORFA Human hg38 2 chr1
NORFA Coelacanth latCha1 2 JH126564
NORFA Elephant loxAfr3 2 scaffold_0
Cow (bosTau9) BLAT Results
BLAT Search Results
ACTIONS QUERY SCORE START END QSIZE IDENTITY CHROM STRAND START END SPAN
browser details NORFA 26 141 166 168 100.0% chr11 - 105959425 105959450 26
browser details NORFA 23 129 154 168 96.2% chr11 + 78399066 78399093 28
browser details NORFA 20 109 128 168 100.0% chr24 + 42274447 42274466 20
Chicken (galGal6) BLAT Results
BLAT Search Results
ACTIONS QUERY SCORE START END QSIZE IDENTITY CHROM STRAND START END SPAN
browser details NORFA 24 120 144 168 100.0% chr3 + 57619879 57619906 28
browser details NORFA 23 119 146 168 96.0% chr14 + 7080696 7080724 29
browser details NORFA 23 119 146 168 96.0% chr14 + 7158580 7158608 29
browser details NORFA 22 120 143 168 95.9% chr2 + 8027210 8027233 24
browser details NORFA 20 66 85 168 100.0% chr25 - 1429916 1429935 20
browser details NORFA 20 127 146 168 100.0% chr1 - 663655 663674 20
Human (hg38) BLAT Results
BLAT Search Results
ACTIONS QUERY SCORE START END QSIZE IDENTITY CHROM STRAND START END SPAN
browser details NORFA 29 58 95 168 94.2% chr3 - 181696143 181696188 46
browser details NORFA 23 124 147 168 100.0% chr17 + 73451838 73451863 26
browser details NORFA 20 138 157 168 100.0% chr8 - 18171443 18171462 20

f

All Genomes BLAT Results

Name	Genome	Assembly	Tiles	Chrom
NORFA	Pig	susScr11	144	chrUn_NW_018084833v1
NORFA	Cow	bosTau9	8	chr11
NORFA	Sheep	oviAri4	6	chr3
NORFA	Chicken	galGal6	4	chr1
NORFA	Human	hg38	4	chr2
NORFA	Mouse	mm10	4	chr16

Cow (bosTau9) BLAT Results

BLAT Search Results

ACTIONS		QUERY	SCORE	START	END	QSIZE	IDENTITY	CHROM	STRAND	START	END	SPAN
browser	details	NORFA	56	50	718	739	49.5%	chr11	+	105958875	105959819	945
browser	details	NORFA	22	301	323	739	100.0%	chr21	-	67144106	67144134	29
browser	details	NORFA	21	313	333	739	100.0%	chr17	-	67110189	67110209	21
browser	details	NORFA	20	576	595	739	100.0%	chr21	+	69527059	69527078	20
browser	details	NORFA	20	164	183	739	100.0%	chr13	+	62677480	62677499	20
browser	details	NORFA	20	6	25	739	100.0%	chr12	+	35225350	35225369	20

Sheep (oviAri4) BLAT Results

BLAT Search Results

ACTIONS		QUERY	SCORE	START	END	QSIZE	IDENTITY	CHROM	STRAND	START	END	SPAN
browser	details	NORFA	92	50	525	739	47.0%	chr3	-	812354	812897	544
browser	details	NORFA	24	1	25	739	100.0%	chr19	-	60265655	60266028	374
browser	details	NORFA	24	434	464	739	44.1%	chr18	+	56438471	56438497	27
browser	details	NORFA	23	189	216	739	88.0%	chr14	-	14071843	14071869	27
browser	details	NORFA	22	441	466	739	92.4%	chr20	+	14229295	14229320	26
browser	details	NORFA	22	2	24	739	100.0%	chr2	+	229784821	229784846	26
browser	details	NORFA	20	191	210	739	100.0%	chr20	-	48691001	48691020	20
browser	details	NORFA	20	164	183	739	100.0%	chr13	+	62004179	62004198	20

Human (hg38) BLAT Results

BLAT Search Results

ACTIONS		QUERY	SCORE	START	END	QSIZE	IDENTITY	CHROM	STRAND	START	END	SPAN
browser	details	NORFA	27	31	58	739	100.0%	chr17	+	33333051	33333088	38
browser	details	NORFA	24	486	510	739	100.0%	chr15	-	47689554	47689596	43
browser	details	NORFA	23	512	535	739	100.0%	chr17	-	73451838	73451863	26
browser	details	NORFA	20	141	160	739	100.0%	chr17	+	10198866	10198885	20
browser	details	NORFA	20	35	56	739	95.5%	chr11	+	40930981	40931002	22

Mouse (mm10) BLAT Results

BLAT Search Results

ACTIONS		QUERY	SCORE	START	END	QSIZE	IDENTITY	CHROM	STRAND	START	END	SPAN
browser	details	NORFA	28	499	539	739	93.8%	chr14	+	11448625	11448672	48
browser	details	NORFA	25	504	539	739	81.5%	chr17	+	46612779	46612811	33
browser	details	NORFA	25	633	658	739	100.0%	chr10	+	60738891	60738922	32
browser	details	NORFA	24	310	334	739	100.0%	chr13	+	69969870	69969913	44
browser	details	NORFA	23	359	382	739	100.0%	chr12	-	71995296	71995331	36
browser	details	NORFA	21	223	243	739	100.0%	chr6	+	64452496	64452516	21
browser	details	NORFA	21	47	69	739	95.7%	chr13	+	17404261	17404283	23

g

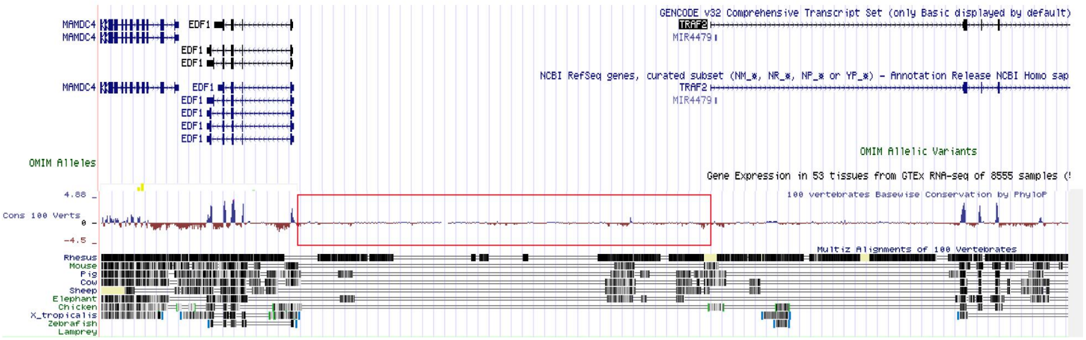

Supplementary Figure 2. Identification of the conservation of pig NORFA structure. (**a**) Diagram depicting the investigation strategy for pig NORFA conservation. (**b**) The secondary structure of pig NORFA was analyzed by RNAfold and RNAstructure software. H: helice, J: junction. (**c**) The conservation of NORFA secondary structure, diagram showing the conservation of H21 of pig NORFA by UCSC database among different species. (**d**) The tertiary structure and domains of pig NORFA were predicted and the conservation of these domains were detected using UCSC. (**e**) The potential ORFs within pig NORFA (sense and anti-sense) were predicted and their conservation were detected by UCSC. (**f**) The conservation of pig NORFA primary structure was detected. (**g**) The conservation of desert region between *EDF1* and *TRAF2* (red box) among different mammal species were detected.

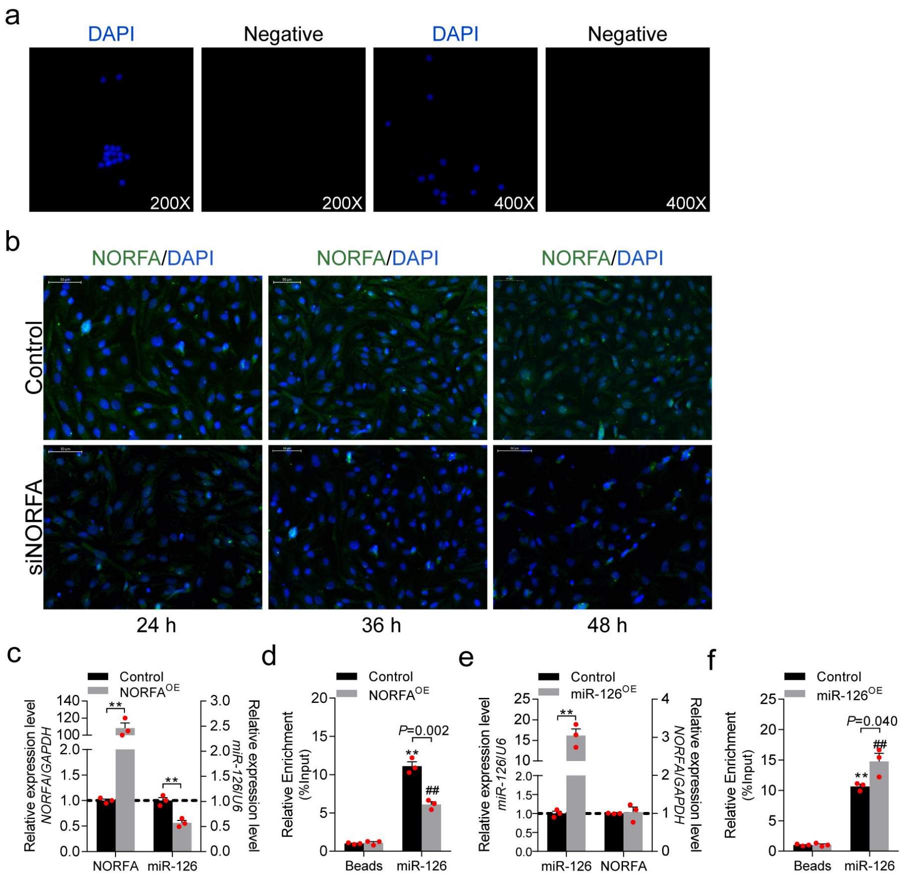

Supplementary Figure 3. NORFA physically interacts with miR-126 in granulosa cells. (**a**) The negative control for RNA FISH assays. (**b**) The signals of *NORFA* in porcine granulosa cells after transfection with *NORFA*-siRNA (si*NORFA*) for different time (24 h, 36 h and 48 h) were detected by FISH assays. (**c**) The expression levels of NORFA and miR-126 in NORFA overexpressed porcine granulosa cells were detected by qRT-PCR. (**d**) The enrichment of miR-126 on biotin-labeled NORFA in NORFA overexpressed porcine granulosa cells was detected by RNA pull-down. (**e**) The expression levels of miR-126 and NORFA in miR-126 overexpressed porcine granulosa cells were detected by qRT-PCR. (**f**) The enrichment of miR-126 on biotin-labeled NORFA in miR-126 overexpressed porcine granulosa cells was detected by RNA pull-down. Data in **c-f** are shown as mean ± S.E.M. with three independent experiments. *P*-values were calculated by a two-tailed Student's *t*-test. $^{**,\#\#}P < 0.01$.

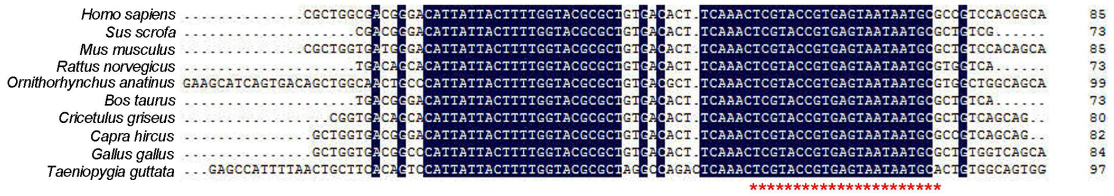

Supplementary Figure 4. Multiple-sequence alignment of pre-miR-126 from different species. The multiple-sequence alignment of pre-miR-126 from 10 different species were shown and asterisks in red indicate the sequence of mature sequence of miR-126.

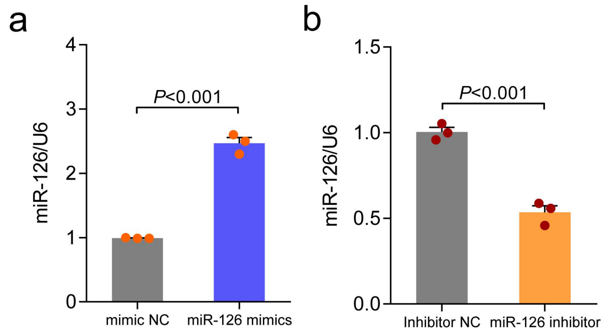

Supplementary Figure 5. Forced expression and silencing of miR-126 in porcine granulosa cells. (**a**) The expression levels of miR-126 were measured by qRT-PCR after porcine granulosa cells transfection with mimics NC (n=3) or miR-126 mimics (n=3). (**b**) qRT-PCR was performed and miR-126 expression levels were detected in porcine granulosa cells transfected with inhibitor NC (n=3) or miR-126 inhibitor (n=3). Data are shown as mean ± S.E.M. by a two-tailed Student's *t*-test.

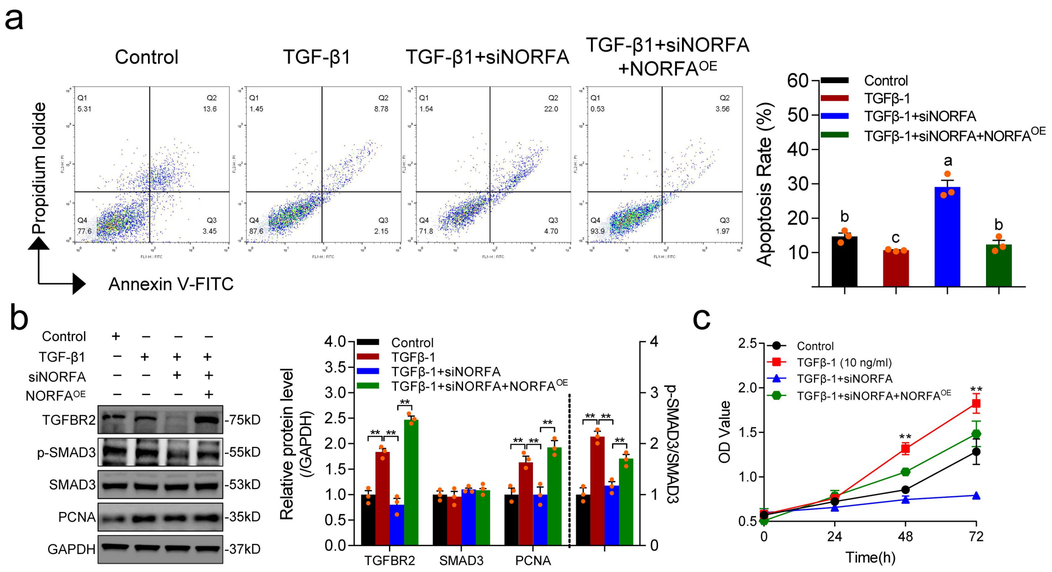

Supplementary Figure 6. NORFA affects the sensitive of porcine granuolasa cells to TGF-β1. (**a**) The apoptosis rate of porcine granulosa cells treated with TGF-β1 and NORFA-siRNA (siNORFA) or/and pcDNA3.1-NORFA ($NORFA^{OE}$) were measured by FACS. (**b**) The protein levels of TGFBR2, p-SMAD3, SMAD3 and PCNA in porcine granulosa cells treated with TGF-β1 and siNORFA or/and $NORFA^{OE}$ were determined by western blotting and normalized to GAPDH. GAPDH was used as a loading control. (**c**) The proliferation levels of porcine granulosa cells treated with TGF-β1 and siNORFA or/and $NORFA^{OE}$ were measured by CCK-8. Data are shown as mean ± S.E.M. with three independent experiments. *P*-values were calculated by a two-tailed Student's *t*-test. $^{**}P < 0.01$.

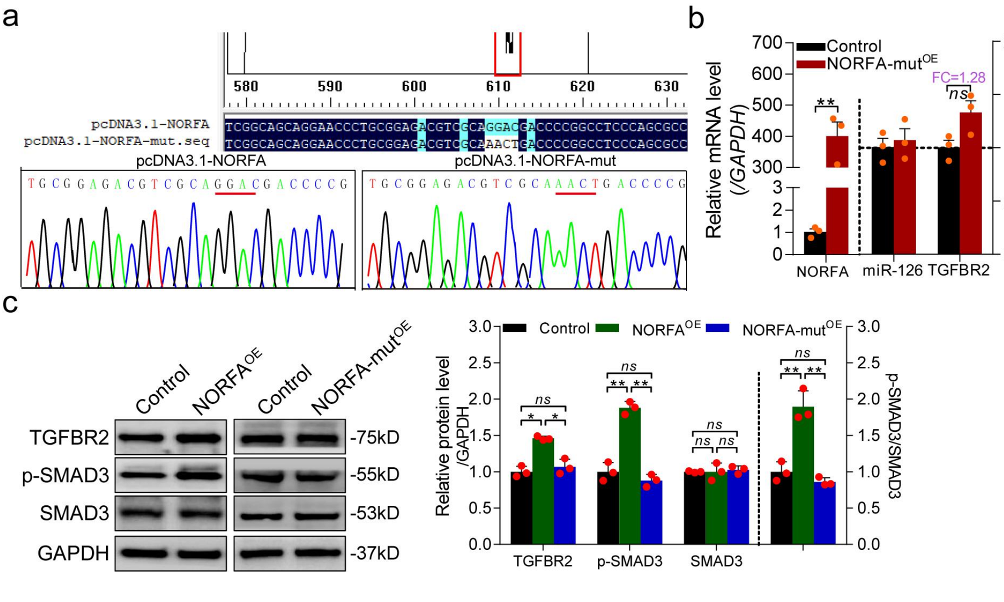

Supplementary Figure 7. NORFA/miR-126/TGFBR2 axis regulates TGF-β signaling pathway in granulosa cells. (**a**) Construction of pcDNA3.1-NORFA-mut vector with miR-126 response element mutation. (**b**) The expression levels of NORFA, miR-126 and TGFBR2 in porcine granulosa cells transfected with pcDNA3.1-NORFA-mut vectors were measured by qRT-PCR. (**c**) The protein levels of TGFBR2 and p-SMAD3 in porcine granulosa cells treated with NORFA^{OE} or NORFA-mut^{OE} were detected by western blot. Data were shown as mean ± SEM with three independent experiments. *P* values were calculated by using a two-tailed Student's t-test. $^{*}P$<0.05, $^{**}P$<0.01 and ns indicates no significance.

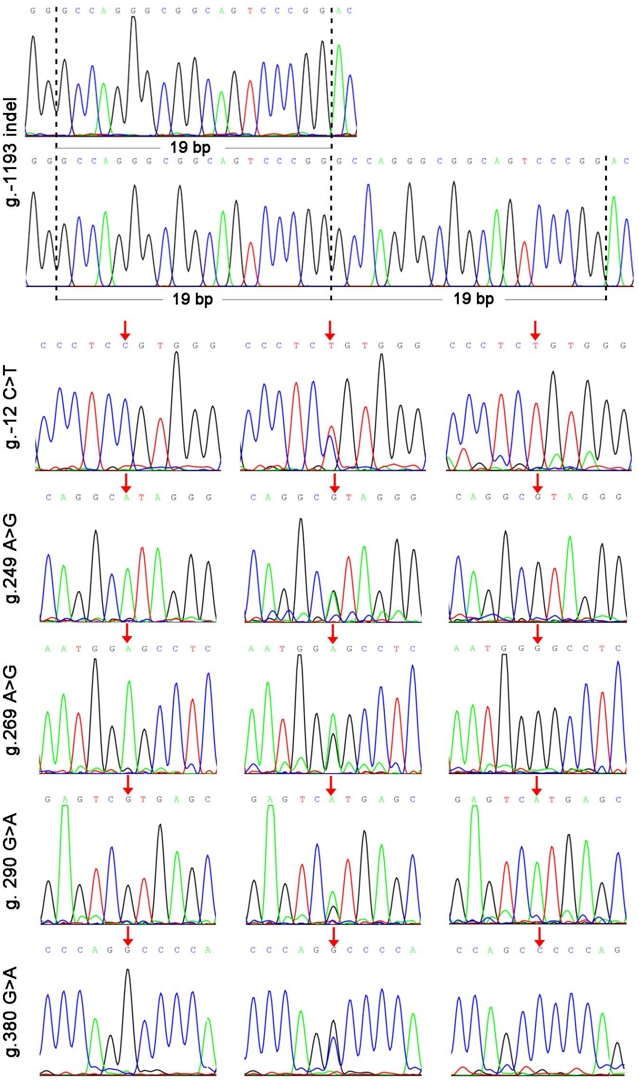

Supplementary Figure 8. Identification of *NORFA* variants in Erhualian, Yorkshire and Landrace sow groups. A 19-bp duplication (g.-1193 indel) and 5 point variants (g.-12C>T, g.249A>G, g.269A>G, g.290G>A and g.380G>A) were identified within *NORFA*. Sanger sequencing traces for these variants were shown and their locations were indicated by red arrows.

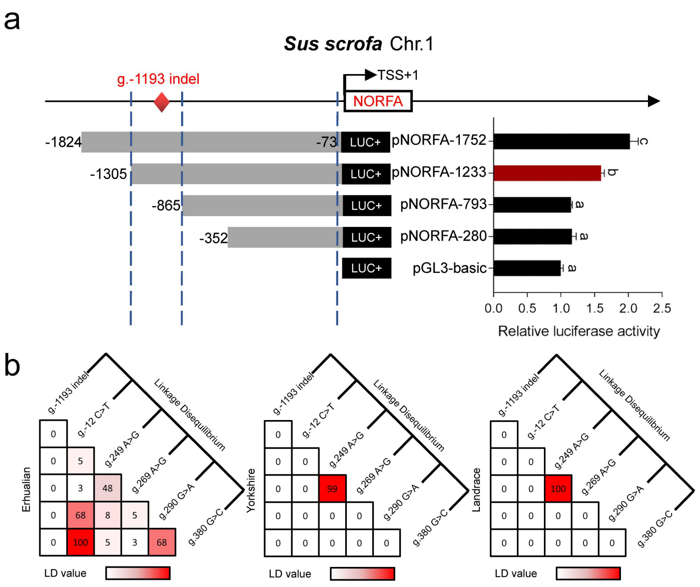

Supplementary Figure 9. Characterization of the 19-bp duplication variant within *NORFA.* (**a**) Schematic showing the luciferase reporters containing different fragments of *NORFA* 5'-flanking sequences. The core promoter of *NORFA* was identified using dual-luciferase activity assay. The transcription start site (TSS) is indicated with black arrow and the 19-bp duplication is represented with red diamond. (**b**) The linkage disequilibrium (LD) analyses. The LD values of these six variants within *NORFA* from Erhuanlian, Yorkshire and Landrace sow groups were calculated by SHEsis software.

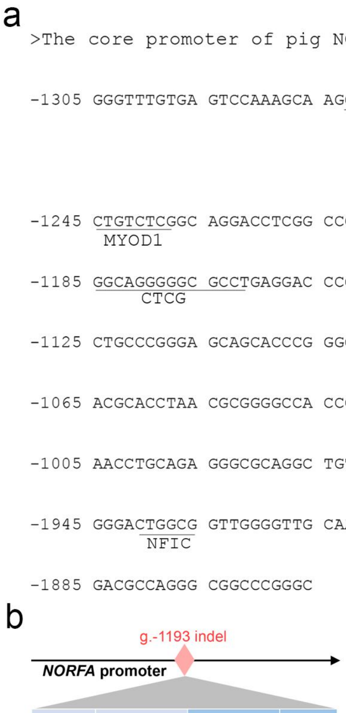

	del/del	ins/ins	
TF	Location	Location	Score
NFIX	-1215/-1206	-1234/-1225	10.687
		-1215/-1206	
E2F6	-1207/-1197	-1226/-1217	6.606
		-1207/-1197	
E2F4	-1208/-1198	-1228/-1217	6.584
		-1208/-1198	
PAX2	-1201/-1194	-1220/-1213	5.548
		-1201/-1194	
GABPA	-1206/-1195	-1225/-1214	5.396
		-1206/-1195	

Supplementary Figure 10. Characterization of the core promoter of porcine *NORFA*. (**a**) The core promoter sequence of *NORFA* containing 19-bp duplication was shown. The binding motifs of the potential transcription factors (TFs) were labeled with underlines. (**b**) The binding motifs of TFs within 19-bp duplication alleles were analyzed by JASPAR. 5 novel binding sites for NFIX, E2F6, E2F4, PAX2 and GABPA were identified, and their locations (red) with prediction scores were represented.

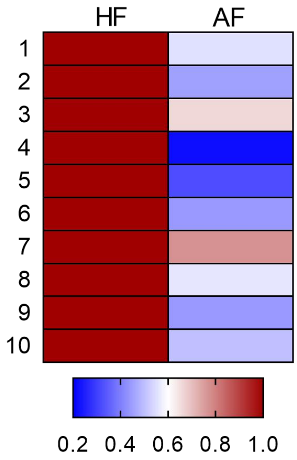

Supplementary Figure 11. NFIX is differentially expressed during follicular atresia. Heatmap showing the mRNA levels of NFIX in porcine granulosa cells from healthy follicles (HF) and atretic follicles (AF), analyzed by qRT-PCR (n=10). The expression levels of NFIX in AF were normalized to that in HF.

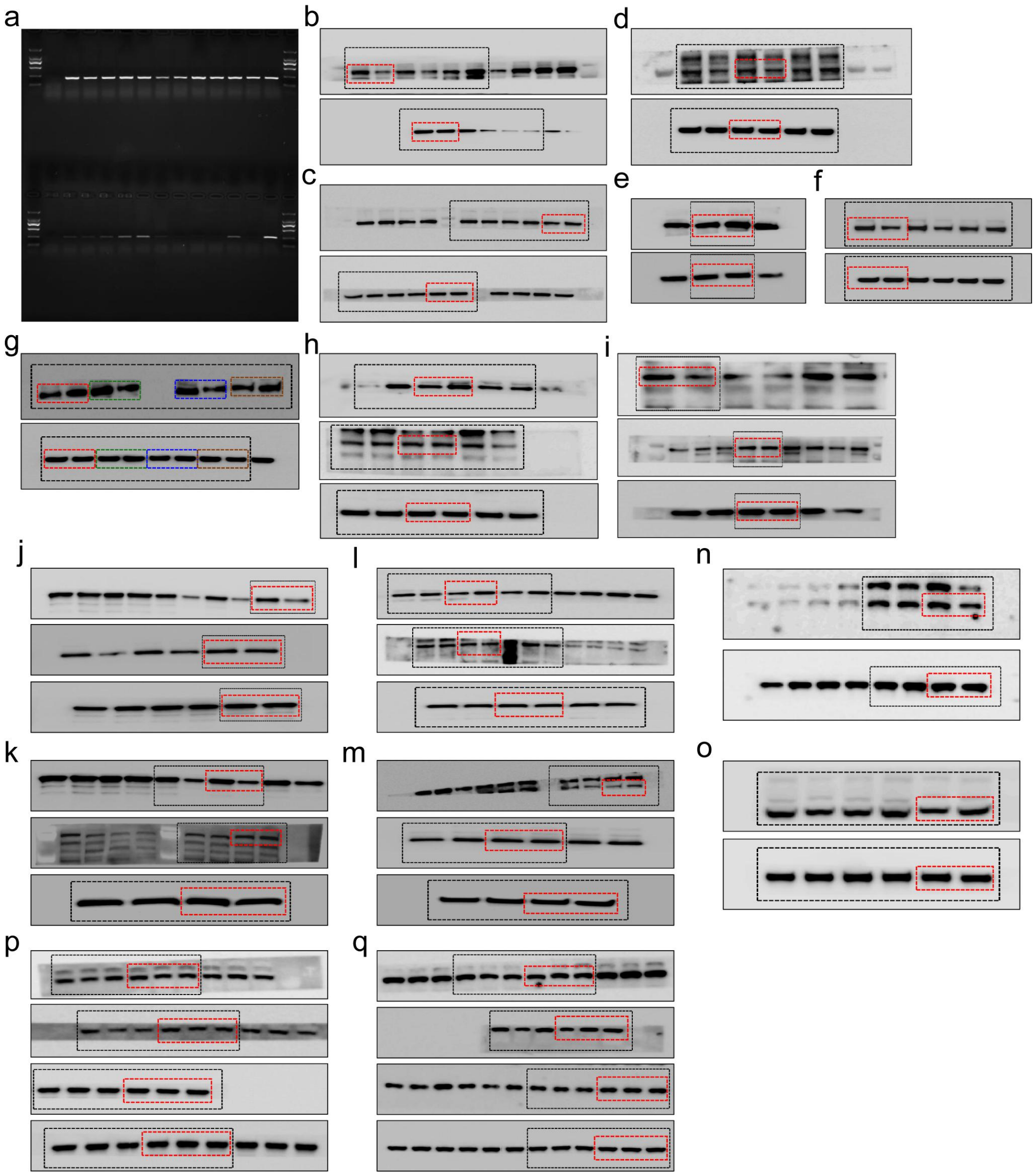

Supplementary Figure 12. The orignal images of gels and blots. (**a**) The orignal gel image for Figure1e. (**b-q**) The orignal blot images in the main figures of this manuscript. Figure6h (**b**), Figure6i (**c**), Figure7b (**d**), Figure7d (**e**), Figure7e (**f**), Figure8d-g (**g**), Figure8h (**h**), Figure8i (**i**), Figure8j (**j**), Figure8k (**k**), Figure8l (**l**), Figure8m (**m**), Figure9e (**n**, **o**), Figure10c (**p**) and Figure10d (**q**). The dotted rectangles in red, green, blue and brown indicated the representative images in the main text and the dotted rectangles in black showed the orignal images involved the corresponding experiments in this research.

Supplementary Tables

Supplementary Table 1 Oligonucleotide sequences used in this study

Name	Sequence (5’ to 3’)	Source
Mimics NC	UUGUACUACACAAAAGUACUG	This study
MiR-126	UCGUACCGUGAGUAAUAAUGCG	This study
Inhibitor NC	CAGUACUUUUGUGUAGUACAA	This study
MiR-126 inhibitor	CGCAUUAUUACUCACGGUACGA	This study
NC-siRNA	Sense: UUCUCCGAACGUGUCACGUTT	This study
	Anti-Sense: ACGUGACACGUUCGGAGAATT	
TGFBR2-siRNA	Sense: GCCAACAACAUCAACCACATT	This study
	Anti-Sense: UGUGGUUGAUGUUGUUGGCTT	
NORFA-siRNA	Sense: CAGACAGAUGUGGAUGAAUTT	This study
	Anti-Sense: AUUCAUCCACAUCUGUCUGTT	
NFIX-siRNA	Sense: CCAACCGGUUUGUCAGCAUTT	This study
	Anti-Sense: AUGCUGACAAACCGGUUGGTT	

Supplementary Table 2. Primers designed for qRT-PCR

Genes	Primer sequence (5'-3')	Usage
miR-126	CCTGTTGTCTCCAGCCACAAAAGAGCACAATATTTCAGGAGAC AACAGGCGCATTA	Reverse-transcription[1]
miR-126	F: CGGGCCACCACCTCCCCTGCAA	qRT-PCR
	R: CAGCCACAAAAGAGCACAAT	qRT-PCR
EGFL7	F: TGCCAGACAGATGTGGATGAA	qRT-PCR
	R: TCCGCTGTTGTGGGGTTT	
AGPAT2	F: CGCTCGTGCGTCATCATCT	qRT-PCR
	R: TCCAGCACCTCCACCTTGAT	
LCN12	F: CGTTTCTTTCCCGCTTCTGT	qRT-PCR
	R: CCTGAGTGTATCCTCCCTGTGC	
C8G	F: CGGACTACCGGAGCTTTGC	qRT-PCR
	R: AGCGTCGCAGAAGCCGTAC	
FBXW5	F: CCAGAATCTCAACGCCAGCAC	qRT-PCR
	R: TGCCGCCCAGGTCAAAGA	
TRAF2	F: AGCCCTTCCTCGGCCAGA	qRT-PCR
	R: GTTCAGCACGCAGACGATGT	
EDF1	F: CGAGCACTACAGTGGCGACTCAGAT	qRT-PCR
	R: GCTCCACTATGCTGGACAGGCAGT	
MAMDC4	F: ACCGTTCGGATGAGGACACA	qRT-PCR
	R: GACCCATGCAGATAGTGATAGAAGA	
PHPT1	F: CCACGGGATTGGTCACAGA	qRT-PCR
	R: ACGGCGTGTACTCGAATCAG	
LCN8	F: CGAGCAATACGCCATCCTG	qRT-PCR
	R: CCGTGTCTGCCGTCAACTC	
LCN5	F: CTGGTACGAGATCGCCTTGG	qRT-PCR
	R: ACGATGGCGTAGGTCCTGTA	
LCN6	F: GACCGCCCTTCTGGCTTTG	qRT-PCR
	R: GATGGTTCTGCGGCGTGAG	
LCN10	F: TGTCAGGCGGCTCTGTGCTT	qRT-PCR
	R: CTGAAGAGCAGCAGCGTCTTG	
FAM69B	F: TTCCGCAAAGGCATCATCTCA	qRT-PCR
	R: TGCCCTGCCAGAGCCCACT	
TMEM141	F: ATCAGGCTGTCTCACGCTCTT	qRT-PCR
	R: CCGTCGCCTGTGATTGGTAG	
CCDC183	F: CCAAGAACAAGGCGACGAT	qRT-PCR
	R: ACGCGGTCGAAGACGTACTT	
BAX	F: CCGAAATGTTTGCTGACG	qRT-PCR
	R: AGCCGATCTCGAAGGAAGT	
BCL2	F: TTCTTTGAGTTCGGTGGGG	qRT-PCR
	R: CCAGGAGAAATCAAATAGAGGC	
TGFBR2	F: GTGCCCAAGCAGGTCATTCA	qRT-PCR
	R: CTCCTCAGGGCTTCGGTCA	
GAPDH	F: GATGGTGAAGGTCGGAGTG	qRT-PCR
	R: CGAAGTTGTCATGGATGACC	
U6	F: TTATGGGTCCTAGCCTGAC	qRT-PCR
	R: CACTATTGCGGGTCTGC	
Linc-NORFA	F: AGGAACCTGCGTGGGAATC	qRT-PCR
	R: CCGATCTCCCAGCATGAAA	
Rplp0	F: TCCAGGCTTAGGCATCACC	qRT-PCR
	R: GGCTCCCACTTTGTCTCCAG	
NFIX	F: CGAGGAGCGAGCGGTGAA	qRT-PCR
	R: TGGCGAAGGCAGTCAATCC	

[1]. Stem-loop reverse-transcriptional primers designed for detect the expression level of mature miRNAs.
F: Forward primers; R: Reverse primers.

Supplementary Table 3. Primers used for plasmids construction

Plasmids	Primer sequence (5'-3')	Vector	Usage
NORFA-MRE-wt	F: CCTGGGAAGCAAGACTTTGTC	*pmirGLO*	vector
	R: GGAGGCTGAGTTTGCCACA		construction
NORFA-MRE1-mut	F: AAAATGTGTTTGGTATGAGCGCGCTGAGATTGGCAGTCC	*pmirGLO*	Mutation
	R: GGGGGCGGAGCGCTGGGGACAGGGAGCTTGGCAAGG		
NORFA-MRE2-mut	F: AGGAACCCTTTGGAGGTGGTGTATTGTTGCCGGCCTC	*pmirGLO*	Mutation
	R: GCTGCCGAGGGGAGCTTCACCTGCAGCACTGCACCCCT		
TGFBR2-MRE-wt	F: TGACCTATAGGACTTGCTCGGA	*pmirGLO*	vector
	R: CACCTTCACTGTGTGCTAAAATT		construction
TGFBR2-MRE-mut	F: TCTCTGATGTCTTCTCCGCGCGCGATTGGGACCGTGCATT	*pmirGLO*	Mutation
	R: AACCCAACATGAGAGACCATCTATGACATAACTGGTCTGAA		
pNORFA-1752	F: CGGGGTACCGGTCGTCATGGAATATGCTCG	*pGL3-basic*	
	R: CCGCTCGAGCCTCCCAATACGATCTACTTCCTG		
pNORFA-1233	F: CGGGGTACCGGGTTTGTGAGTCCAAAGCAA	*pGL3-basic*	
	R: CCGCTCGAGCCTCCCAATACGATCTACTTCCTG		
pNORFA-793	F: CGGGGTACCACCTGTAATCCTGCTGGTGAGA	*pGL3-basic*	vector
	R: CCGCTCGAGCCTCCCAATACGATCTACTTCCTG		construction
pNORFA-280	F: CGGGGTACCCCAGCATCGCGTCGTCTTT	*pGL3-basic*	
	R: CCGCTCGAGCCTCCCAATACGATCTACTTCCTG		
pcDNA3.1-NFIX	F: GCCCCCAACTCACAACTCTG	*pcDNA3.1*	
	R: GGGGATTTTTCCACGTCTCAA		

F: Forward primers; R: Reverse primers.

Supplementary Table 4. Primers used for ChIP assay

Primers	Sequence (5’-3’)	Temp	Product size
NORFA	F: GCAAAGTCCCCACGGAGACCA	62 ℃	139 bp for A1/A1
	R: AGCCTGGCGGGTCCTCAGGC		120 bp for A2/A2
SBEX	F:CAGCAAAGGGTGGCAAGGT	60 ℃	145 bp
	R:ATGCCTGTGCCACTTTCAACT		

Supplementary Table 5. Primers used for NORFA polymorphism detection

Primers	Sequence (5'-3')	Annealing temp	Product size
NORFA-P1	F: TGGTCCAGGAGGGCAAGC	62 ℃	637 bp
	R: CCCCGTTTGCGTGCTTGT		
NORFA-P2	F: GGGTTTGTGAGTCCAAAGCAAG	60 ℃	796 bp
	R: GGTTTCCACGTCCCTCGGTAT		
NORFA-P3	F: TTCCAGAGCCTCCAAAGTGA	62 ℃	730 bp
	R: CCTTTGGGAGACCAGCACA		
NORFA-P4	F: GCAGGAAGTAGATCGTATTGGG	62 ℃	630 bp
	R: GGGATTCCCACGCAGGTT		
NORFA-P5	F: CGTAGGGAGCAGCTAATGGG	62 ℃	840 bp
	R: CCAGCTCTAACGGACCTAAACG		
NORFA-P6	F: CCTGGGAAGCAAGACTTTGTC	61 ℃	539 bp
	R: GCAGGCTTTGAAGTTCTGTGATT		

Supplementary Methods

FACS analysis

The apoptosis rate of porcine granulosa cells were assessed by FACS, as described in the methods with Annexin V-FITC and PI dyes. For gating strategy, cells without dye label and two single dye label were respectively usd to detect the FSC/SSC gates, which is shown in Reference 24. Q1 mainly includes the dead cells, Q2 includes the late apoptotic cells, Q3 mainly includes early apoptotic cells, and Q4 mainly includes healthy cells. Besides, the right part of FSC gating (Q2+Q3) was considered as postive apoptotic cells.
