# Supplementary material for: NORFA, long intergenic noncoding RNA, maintains sow fertility by inhibiting granulosa cell death
Source: Commun Biol. 2020 Mar 18;3:131. doi: 10.1038/s42003-020-0864-x (PMC7080823; doi:10.1038/s42003-020-0864-x)
Supplement: Supplementary file 2 — Description of Additional Supplementary Files [file 42003_2020_864_MOESM2_ESM.pdf]

Description of Additional Supplementary Files

Supplementary Data 1: Source data.

All source data underlying the graphs and charts in the main figures are available in Supplementary Data 1.
