# Supplementary material for: NORFA, long intergenic noncoding RNA, maintains sow fertility by inhibiting granulosa cell death
Source: Commun Biol. 2020 Mar 18;3:131. doi: 10.1038/s42003-020-0864-x (PMC7080823; doi:10.1038/s42003-020-0864-x)
Supplement: Supplementary file 4 — Reporting Summary [file 42003_2020_864_MOESM4_ESM.pdf]

nature research

Corresponding author(s): Qifa Li

Last updated by author(s): Feb 13, 2020

Reporting Summary

Nature Research wishes to improve the reproducibility of the work that we publish. This form provides structure for consistency and transparency in reporting. For further information on Nature Research policies, see Authors & Referees and the Editorial Policy Checklist.

Statistics

For all statistical analyses, confirm that the following items are present in the figure legend, table legend, main text, or Methods section.

n/a	Confirmed	
☐	☒	The exact sample size (*n*) for each experimental group/condition, given as a discrete number and unit of measurement
☐	☒	A statement on whether measurements were taken from distinct samples or whether the same sample was measured repeatedly
☐	☒	The statistical test(s) used AND whether they are one- or two-sided<br>*Only common tests should be described solely by name; describe more complex techniques in the Methods section.*
☐	☒	A description of all covariates tested
☒	☐	A description of any assumptions or corrections, such as tests of normality and adjustment for multiple comparisons
☐	☒	A full description of the statistical parameters including central tendency (e.g. means) or other basic estimates (e.g. regression coefficient) AND variation (e.g. standard deviation) or associated estimates of uncertainty (e.g. confidence intervals)
☐	☒	For null hypothesis testing, the test statistic (e.g. *F*, *t*, *r*) with confidence intervals, effect sizes, degrees of freedom and *P* value noted<br>*Give P values as exact values whenever suitable.*
☒	☐	For Bayesian analysis, information on the choice of priors and Markov chain Monte Carlo settings
☒	☐	For hierarchical and complex designs, identification of the appropriate level for tests and full reporting of outcomes
☐	☒	Estimates of effect sizes (e.g. Cohen's *d*, Pearson's *r*), indicating how they were calculated

Our web collection on statistics for biologists contains articles on many of the points above.

Software and code

Policy information about availability of computer code

Data collection	Microsoft Excel, ImageJ (NIH), StepOne Plus Real-Time PCR system (ThermoFisher Scientific), GloMax detection system (Promega), BD FACSCalibur cytometry (Becton Dickinson), Nikon Eclipse 80i microscope with a Nikon DS-2 digital camera (Nikon), Image Lab (Bio-Rad).
Data analysis	Microsoft Excel, ImageJ (NIH), Flowjo software v10.0 (TreeStar), IBM SPSS Statistics v20.0 (SPSS Inc.), Graphpad v7.0 (Graphpad).

For manuscripts utilizing custom algorithms or software that are central to the research but not yet described in published literature, software must be made available to editors/reviewers. We strongly encourage code deposition in a community repository (e.g. GitHub). See the Nature Research guidelines for submitting code & software for further information.

Data

Policy information about availability of data

All manuscripts must include a data availability statement. This statement should provide the following information, where applicable:

- Accession codes, unique identifiers, or web links for publicly available datasets
- A list of figures that have associated raw data
- A description of any restrictions on data availability

Our data in this study are available within the paper.

Field-specific reporting

Please select the one below that is the best fit for your research. If you are not sure, read the appropriate sections before making your selection.

☒ Life sciences ☐ Behavioural & social sciences ☐ Ecological, evolutionary & environmental sciences

Life sciences study design

All studies must disclose on these points even when the disclosure is negative.

Sample size	For in vitro experiments: n=3 for gene expression and protein levels, luciferase activities, FACS for apoptosis detection assay in cultured cells. As for in vivo studies: n=7 for gene expression and n=3 for protein levels, n=3 for histological and FISH assays, n=10 for Pearson correlation analysis.
Data exclusions	No data was excluded from the analyses in this study.
Replication	In this study, three independent experiments were replicated t verify the findings and all the attempts were successful.
Randomization	The porcine granulosa cells from different ovaries were collected and mixed up, then they were randomly seeded into culture plates. Group in culture plates were equally arranged.
Blinding	The samples were encrypted and associated with the code number.

Reporting for specific materials, systems and methods

We require information from authors about some types of materials, experimental systems and methods used in many studies. Here, indicate whether each material, system or method listed is relevant to your study. If you are not sure if a list item applies to your research, read the appropriate section before selecting a response.

Materials & experimental systems

n/a	Involved in the study	
☐	☒	Antibodies
☐	☒	Eukaryotic cell lines
☒	☐	Palaeontology
☐	☒	Animals and other organisms
☒	☐	Human research participants
☒	☐	Clinical data

Methods

n/a	Involved in the study	
☒	☐	ChIP-seq
☐	☒	Flow cytometry
☒	☐	MRI-based neuroimaging

Antibodies

Antibodies used	Anti-TGFBR2 rabbit polyclonal (Sangon Biotech, Lot #D155818); Anti-SMAD3 rabbit polyclonal (Sangon Biotech, Lot #D155234); Anti-p-SMAD3 rabbit polyclonal (Sangon Biotech, #155153); Anti-NFIX rabbit polyclonal (Affinity, Lot #DF3250); Anti-GAPDH mouse polyclonal (ORIGENE, Lot #TA802519); Donkey anti-rabbit IgG-HRP (Santa Cruz Biotechnoloty, Cat #sc2077, Lot #K0810); Horse anti-mouse IgG-HRP (Cell Signaling Technology, Lot #7076).
Validation	Anti-TGFBR2 rabbit polyclonal, anti-SMAD3 rabbit polyclonal and anti-p-SMAD3 rabbit polyclonal (Specificity, human, mouse, rat and have been validated by previous studies in pig which referred in this study or have the validation statements on the manufacturer's websites; Tested application, WB, IHC). Anti-NFIX rabbit polyclonal (Specificity, human, mouse and pig; Tested application, WB, IHC, ELISA and IP). Anti-GAPDH mouse polyclonal (Specificity, mouse and has been validated by previous studies; Tested application, WB, IF, IHC).

Eukaryotic cell lines

Policy information about cell lines

Cell line source(s)	HEK 293T cells, Nanjing Medical University (Dr. Jiying Liu, China).
Authentication	Authenticated by Dr. Jiying Liu and Nanjing Medical University.
Mycoplasma contamination	Cell line used was tested and showed negative for contaminations.
Commonly misidentified lines (See ICLAC register)	N/A

Animals and other organisms

Policy information about studies involving animals; ARRIVE guidelines recommended for reporting animal research

Laboratory animals	N/A

Wild animals	In this study, a total of 344 sows were random selected. The breeds, numbers and source were provided in the "Materials and methods" section of the manuscript. The sows were breed in the pigsty and transported to slaughterhouse for pork production and ovraies collection. And we also claimed that all the process were performed with less stress and animal welfare.
Field-collected samples	The fresh ovaries were collected at room temperature (~22 ℃) under the circumstance relative humidity 55%-70% and immediately placed in the 37 ℃ PBS.
Ethics oversight	Animal experiments were approved and over sighted by Animal Ethics Committee at Nanjing Agricultural University, China (SYXK (Su) 2015-0656) and performed in accordance with the Regulations for the Administration of Affairs Concerning Experimental Animals (No.2 of the State Science and Technology Commission, 11/14/1988).

Note that full information on the approval of the study protocol must also be provided in the manuscript.

Flow Cytometry

Plots

Confirm that:

☒ The axis labels state the marker and fluorochrome used (e.g. CD4-FITC).

☒ The axis scales are clearly visible. Include numbers along axes only for bottom left plot of group (a 'group' is an analysis of identical markers).

☒ All plots are contour plots with outliers or pseudocolor plots.

☒ A numerical value for number of cells or percentage (with statistics) is provided.

Methodology

Sample preparation	Porcine granulosa cells were collected from healthy follicles and cultured in plates for 48 h. After treatment or transfection for 48 h, granulosa cells were washed by 37 °C PBS twice and digested by typsin without EDTA. After 1,000 rpm centrifugation for 5 mins, cells were collected and resuspended with PBS at 4°C, and repeat twice. Granulosa cells were resuspended with 100 μL binding buffer for 10 mins. In dark room, the cells were incubated with 5 μL Annexiin V-FITC and 5μL PI dyes for another 10 mins. Finally, the resuspended cells (4x10^5 cells in 100 μL of binding buffer) were collected and sent for apoptosis detection.
Instrument	BD FACSCalibur cytometry (Becton Dickinson)
Software	BD FACSDiva (Becton Dickinson Biosciences)
Cell population abundance	According to the References
Gating strategy	According to the References and see more details in Supplementary Information

☒ Tick this box to confirm that a figure exemplifying the gating strategy is provided in the Supplementary Information.
